# Synthesis, crystal structure and Hirshfeld surface analysis of 2,2-di­chloro-3,3-dieth­oxy-1-(4-fluoro­phen­yl)propan-1-ol

**DOI:** 10.1107/S2056989025002154

**Published:** 2025-04-29

**Authors:** Saadet N. Guseynova, Aida I. Samigullina, Cemile Baydere Demir, Necmi Dege, Nayim Sepay, Ennio Zangrando, Khudayar I. Hasanov, Alebel N. Belay

**Affiliations:** a Kosygin State University of Russia, 117997 Moscow, Russian Federation; b N. D. Zelinsky Institute of Organic Chemistry, Russian Academy of Sciences, 119991 Moscow, Russian Federation; c Physiotherapy Program, Vocational School of Health Services, Demiroglu Bilim University, 34570-Istanbul, Türkiye; d Department of Physics, Faculty of Sciences, Ondokuz Mayıs University, Samsun 55200, Türkiye; e Department of Chemistry, Lady Brabourne College, Kolkata 700017, India; f Department of Chemical and Pharmaceutical Sciences, University of Trieste, 34127 Trieste, Italy; g Azerbaijan Medical University, Scientific Research Centre (SRC), A. Kasumzade St. 14, AZ 1022, Baku, Azerbaijan; h Department of Chemistry, Bahir Dar University, PO Box 79, Bahir Dar, Ethiopia; Harvard University, USA

**Keywords:** crystal structure, di­chloro­hydrin, ketoacetal, β-oxo­aldehyde, Hirshfeld surface analysis

## Abstract

The title mol­ecule, C_13_H_17_Cl_2_FO_3_, crystallizes in the ortho­rhom­bic space group *P*2_1_2_1_2_1_ with one mol­ecule in the asymmetric unit. The skeleton of the mol­ecule exhibits an *anti* conformation with a C—C—C—C(Ph) torsion angle of −174.97 (18)°. The species are weakly hy­dro­gen bonded to form a polymeric chain elongated in the direction of the *b* axis. This inter­action is realised by the hydroxyl group with an ether O atom of a symmetry-related species [O—H⋯O hy­dro­gen-bond distance of 2.975 (2) Å]. No π-stacking inter­action involving the fluoro­benzyl moiety is detected in the crystal structure. Hirshfeld surface analysis, confirming the O—H⋯O donor–acceptor inter­actions, indicates that the most important contributions to the surface contacts are H⋯H (47.0%), Cl⋯H (19.5%), C⋯H (12.1%) and F⋯H (10.7%).

## Abstract 2

We have isolated and structurally chracterized 2,2-di­chloro-3,3-dieth­oxy-1-(4-fluoro­phen­yl)propan-1-ol by simple hy­dro­genation of 2,2-di­chloro-3,3-dieth­oxy-1-(4-fluoro­phen­yl)propan-1-one. Hirshfeld surface analysis was performed.

## 1. Chemical context

α-Haloketones and their derivatives are versatile synthetic precursors or building blocks for the synthesis of heterocyclic com­pounds, multidentate ligands, supra­molecular syn­thons, *etc*. (Erian *et al.*, 2003 ▸; Guseinov *et al.*, 2017 ▸, 2022 ▸). We have recently isolated 20-membered macrocycles by the simple condensation of α,α-dihalo-β-oxo­aldehydes with di­amino­furazan in aceto­nitrile, where the inter­ior and exterior sites of these macrocycles com­prise hy­dro­gen- and halogen-bond-donor sites, respectively (Guseinov *et al.*, 2024 ▸). On the other hand, the hy­dro­genation of ketones is an emerging area in synthetic organic chemistry and catalysis, of significant inter­est to the pharmaceutical industry, agrochemicals, *etc*. (Yang & Fang, 2023 ▸). Similar to other supra­molecular systems (Gurbanov *et al.*, 2020 ▸, 2022 ▸), we believe that the halogen atoms in hy­dro­genation products of ketones can act as halogen-bond-donor centres in crystal engin­eering.
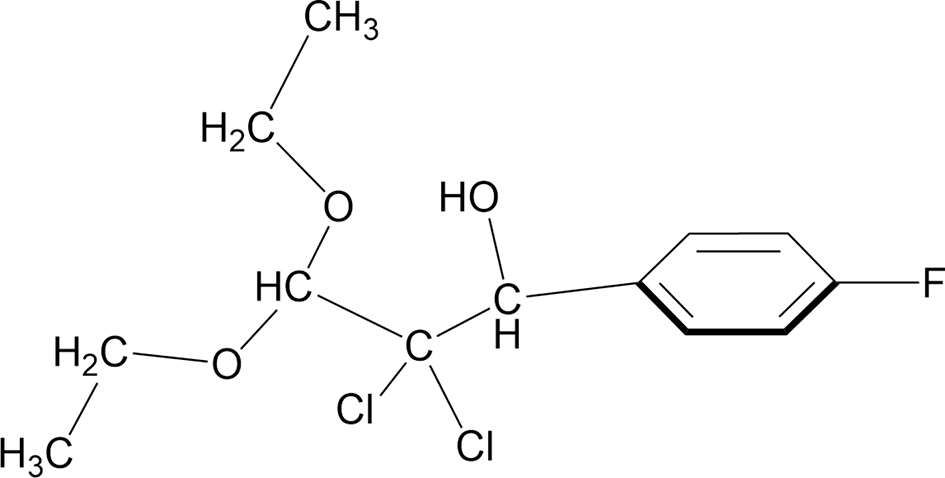


A series of crystal structures of com­pounds obained by reduction of 2,2,2-tri­chloro-1-aryl­ethano­nes by *R*Mg*X*, having a Ph–CHOH–CCl_2_– fragment, have also been reported (Essa *et al.*, 2013 ▸, 2105). Herein, we have used a simple method for the hy­dro­genation of 2,2-di­chloro-3,3-dieth­oxy-1-(4-fluoro­phen­yl)propan-1-one to prepare the title mol­ecule, **1**.

## 2. Structural commentary

**Figure 1:**
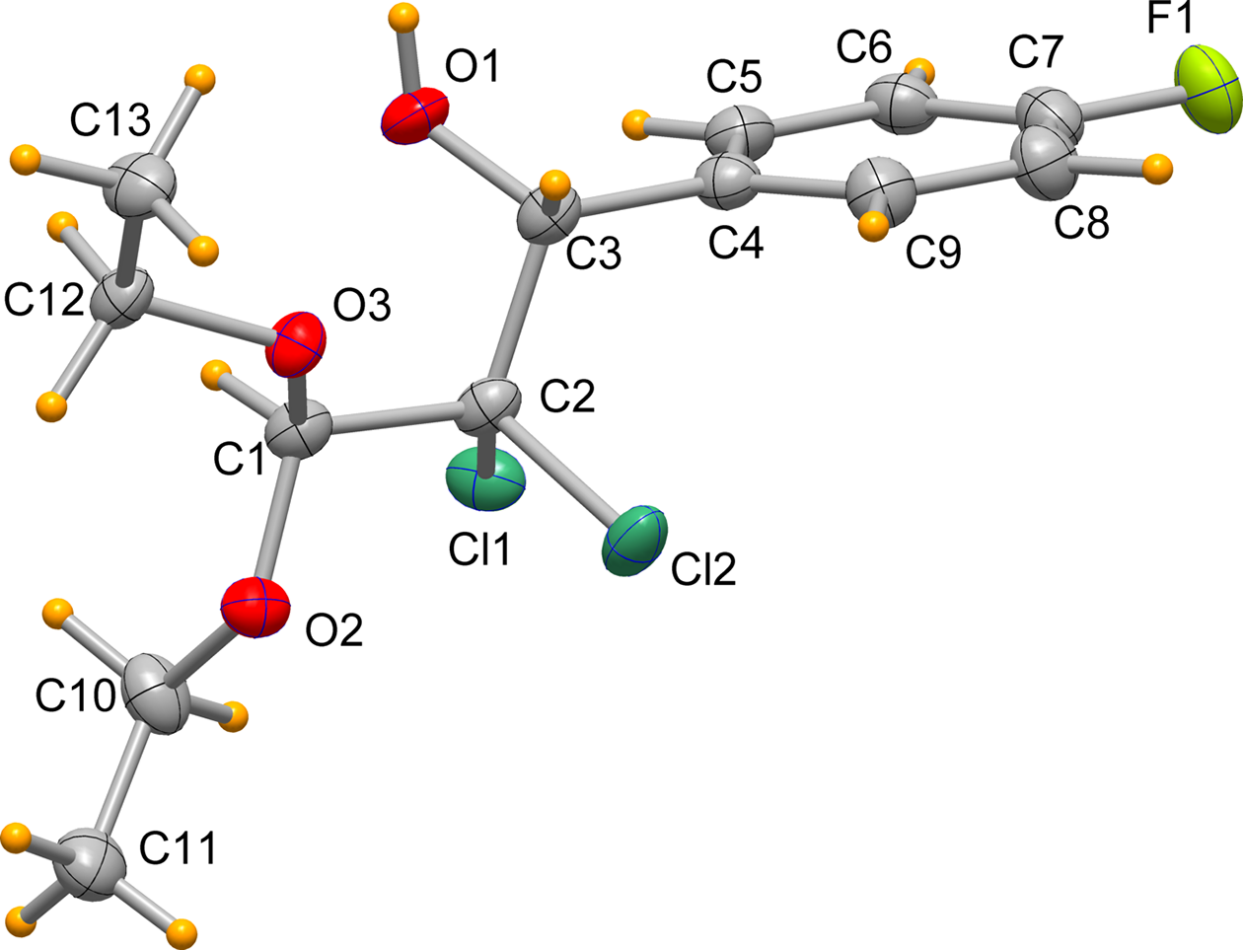
The mol­ecular structure of the asymmetric unit of com­pound **1**. Displacement ellipsoids are drawn at the 50% probability level.

The mol­ecule of the title com­pound (**1**) is shown in Fig. 1 ▸. The central chain with atoms C1, C2, C3 and C4 shows a staggered conformation, with a torsion angle about C2—C3 of 174.97 (18)°. The C2—Cl bond lengths are 1.792 (2) and 1.778 (2) Å, to be com­pared with the value of 1.798 Å reported by Negrier *et al.* (2002 ▸) for 2,2-di­chloro­propane and to the range of 1.791 (1)–1.800 (1) Å measured by Cornia *et al.* (2012 ▸) in a series of 2,2-di­chloro­butan-1-one derivatives. The C1—O(eth­oxy) bond lengths are 1.410 (3) and 1.392 (3) Å. The mol­ecule shows intra­molecular nonconventional hy­dro­gen bonds C5—H5⋯O1 and C10—H10*B*⋯Cl1, with distances between the donor and acceptor atom of 2.820 (3) and 3.326 (3) Å, respectively.

It is worth noting that the mentioned hy­dro­genation reaction for the preparation of the title mol­ecule produced a racemic mixture of mol­ecules, but the crystallization process separated the two chiral isomers. The present mol­ecule crystallizes in the space group *P*2_1_2_1_2_1_ and shows an absolute configuration of *R* at atom C3. The Flack parameter of the structural model determined by the single-crystal structure analysis is −0.001 (6).

## 3. Supra­molecular features

**Figure 2:**
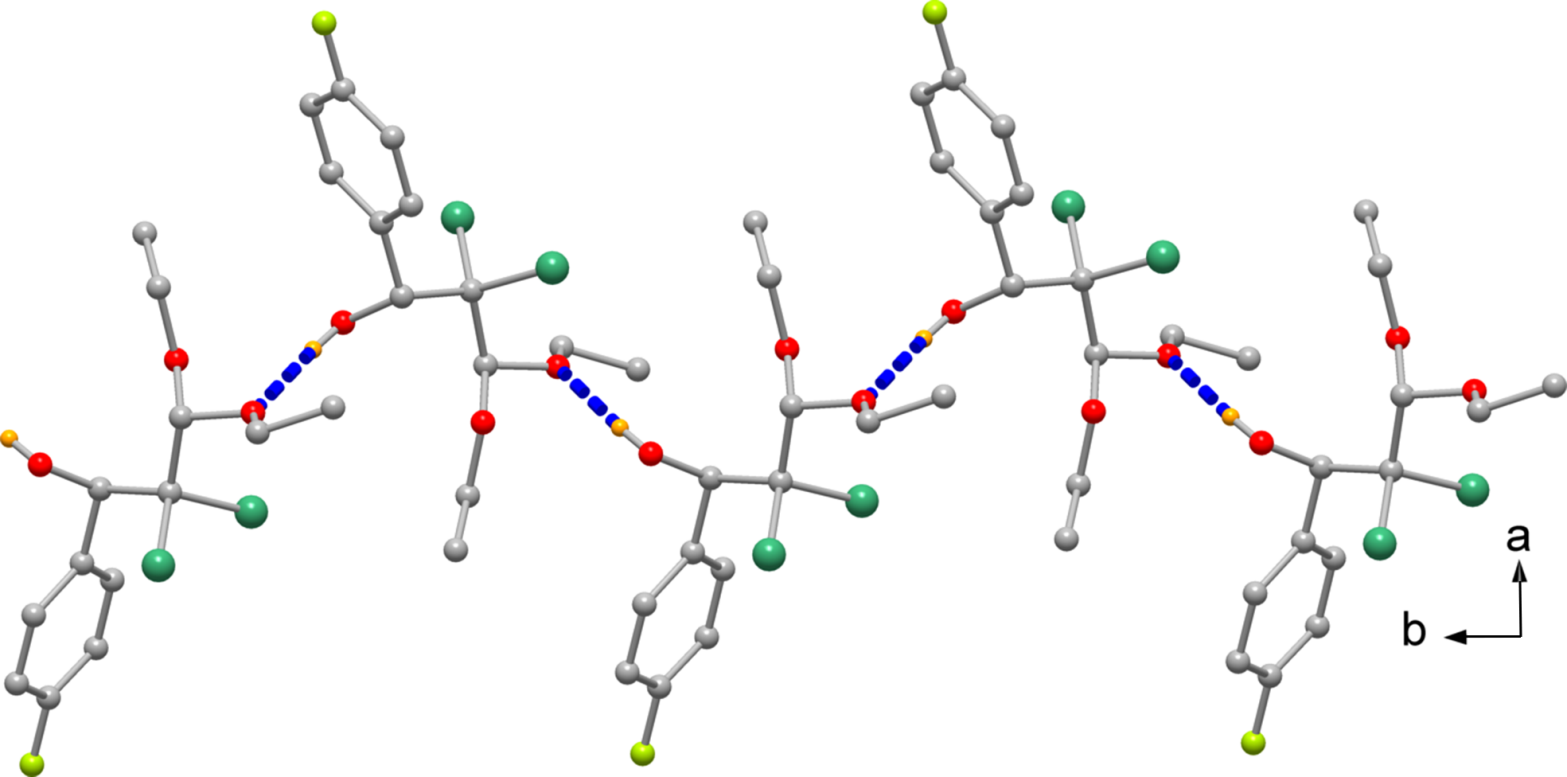
Mono-periodic array built by hy­dro­gen bonds developed in the direction of the *b* axis.

**Figure 3:**
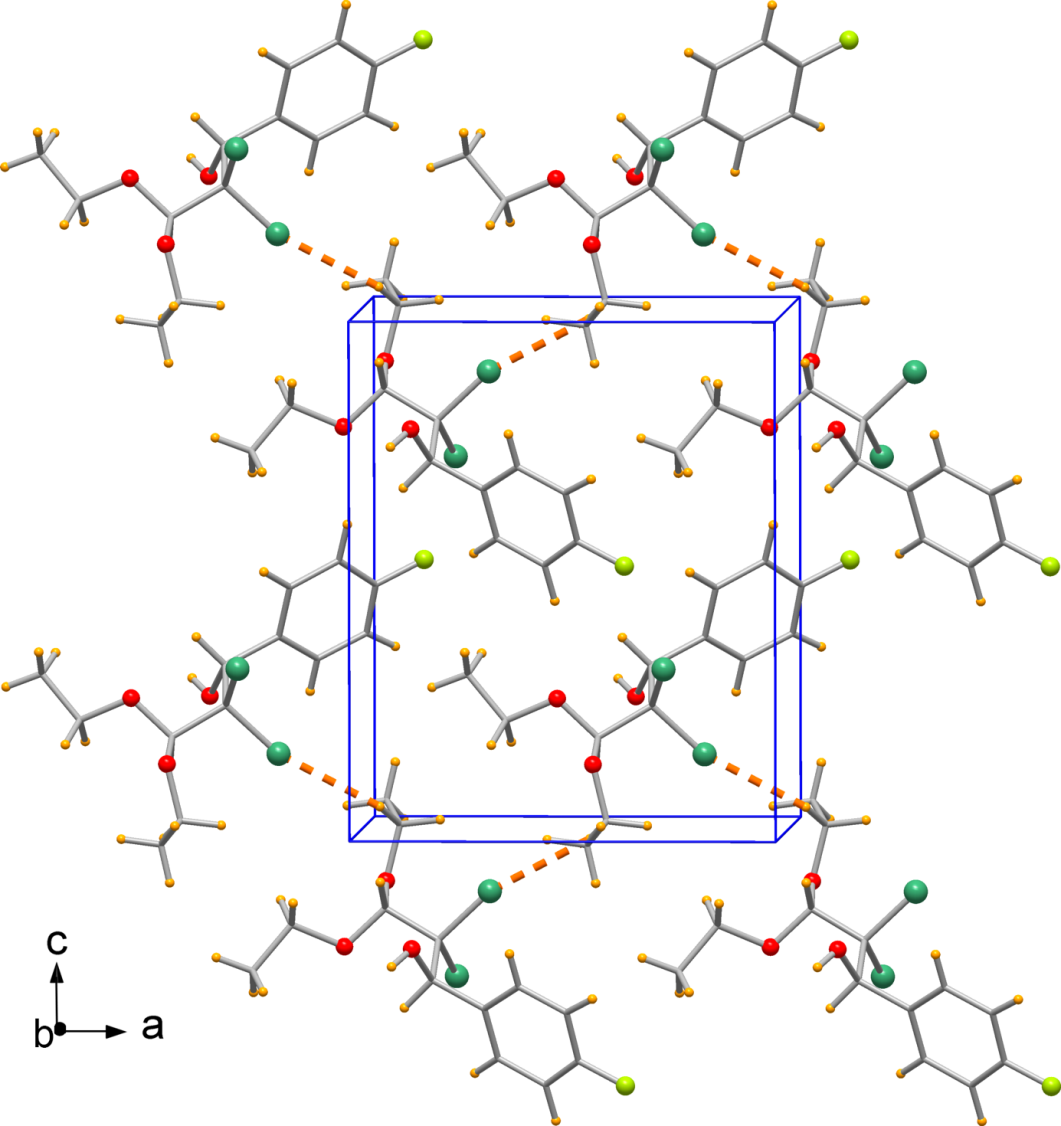
The packing of mol­ecules connected by nonconventional C10—H10*A*⋯Cl1 hy­dro­gen bonds.

The species are hy­dro­gen bonded to form a linear polymeric chain elongated in the direction of crystallographic *b* axis (Fig. 2 ▸ and Table 1 ▸). The O1—H1⋯O2^i^ hy­dro­gen bond has an O⋯O distance of 2.975 (2) Å, while a possible O1—H1⋯O3^i^ inter­action having an O⋯O distance of 3.046 (2) Å is not to be excluded although weaker. In addition, the packing evidences C10—H10*A*⋯Cl1 inter­actions [C⋯Cl distance of 3.636 (3) Å], giving rise to chains developing along the *a* axis (Fig. 3 ▸).

## 4. Hirshfeld surface analysis

**Figure 4:**
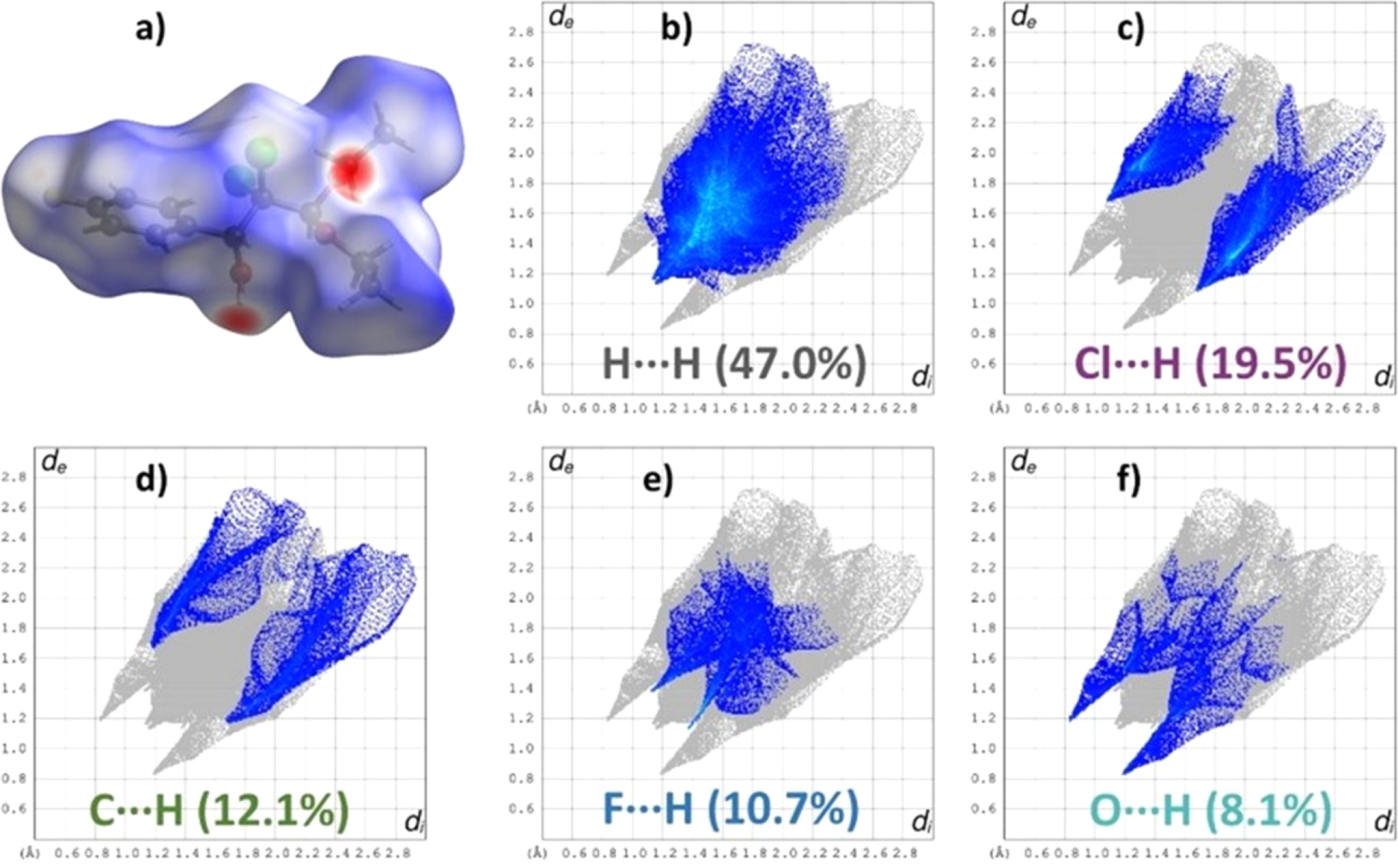
The HS of com­pound **1** over (*a*) *d*_norm_, the *d*_e_*versus d*_i_ plot of the (*b*) H⋯H, (*c*) Cl⋯H, (*d*) C⋯H, (*e*) F⋯H and (*f*) O⋯H inter­actions from the Hirshfeld surface analysis.

The Hirshfeld surface (HS) analysis (Spackman *et al.*, 2009 ▸) identifies and qu­anti­fies noncovalent inter­actions within a crystalline matrix (Biswas *et al.*, 2025 ▸; Das *et al.*, 2025 ▸; Sepay *et al.*, 2023 ▸). Four notable red spots are identified on the surface, specifically on the OH hy­dro­gen, the OH oxygen and the acetal O atoms, indicating substantial donor–acceptor inter­actions, particularly associated with O—H⋯O(π) and C—H⋯O inter­actions [Fig. 4 ▸(*a*)]. Additionally, faint red patches around the F and Cl atoms suggest their minor contributions to the crystal inter­actions. The HS study shows that there are no π–π inter­actions in the solid state.

The HS analysis reveals that the inter­molecular inter­actions in the crystal structure of com­pound **1** are primarily driven by H⋯H inter­actions [Figs. 4 ▸(*b*)–4(*f*)]. A notable spike in the *d*_e_*versus d*_i_ plots was observed at approximately *d*_e_ + *d*_i_ ≃ 2.38 Å, accounting for 47.0% of the total inter­actions [Fig. 4 ▸(*b*)]. The Cl⋯H inter­actions followed, at around 3.32 Å, contributing 19.5% [Fig. 4 ▸(*c*)]. Other weak inter­actions included C⋯H (*d*_e_ + *d*_i_ ≃ 3.26 Å, 12.1%), F⋯H (*d*_e_ + *d*_i_ ≃ 2.78 Å, 10.7%) and Cl⋯F (*d*_e_ + *d*_i_ ≃ 3.6 Å, 1.9%). O⋯H inter­actions, similar to H⋯H inter­actions, showed a spike at 2.38 Å but represented only 8.1% of the total inter­actions [Fig. 4 ▸(*f*)]. The analysis identifies three weak inter­actions, *i.e.* C⋯H, F⋯H and Cl⋯F, affecting the crystallization process. The inter­actions are ranked in importance as H⋯H > Cl⋯H > C⋯H > F⋯H > O⋯H > Cl⋯F, with 12% of inter­actions from O—H⋯C(OEt)_2_ contacts. Total polar inter­actions account for 40.2%, while nonpolar and van der Waals inter­actions make up 59.1%, indicating the amphiphilic nature of the com­pound.

## 5. Database survey

The Cambridge Structural Database (CSD, Version 5.45, update of March 2024; Groom *et al.*, 2016 ▸) was searched for mol­ecules with com­parable groups. The CCl_2_ fragment can be com­pared with that of the mol­ecular structure reports by Negrier *et al.* (2002 ▸; refcode QQQCZS01) describing a study on the polymorphism of 2,2-di­chloro­propane. On the other hand, a study with a series of ketene acetals com­prising a 2,2-di­chloro­butan-1-one fragment was published by Cornia *et al.* (2012 ▸; NEHQIC, NEHQOI, NEHQUO, NEHRAV, NEHREZ, NEHREZ01, NEHROJ and NEHRUP). Of inter­est are structures com­prising a Ph–CHOH–CCl_2_– fragment reported by Essa *et al.* (2013 ▸, 2015 ▸; BETPAT, BETPEX, BETPIB, UHIQOT, UHIQUZ, UHIRAG and UHISOV).

## 6. Synthesis and crystallization

A solution of 2,2-di­chloro-3,3-dieth­oxy-1-(4-fluoro­phen­yl)propan-1-one (3.09 g, 0.01 mol) in methanol (30 ml) was cooled to 263 K. After addition of sodium borohydride (0.4 g, 0.011 mol), the mixture was stirred for 2 h at room tem­per­a­ture. Distilled water (20 ml) and concentrated hydro­chloric acid (1 ml) were then added to the resulting solution and the organic solvent was distilled off on a rotary evaporator. The resulting suspension was extracted with diethyl ether twice and the organic fractions were combined and dried over anhydrous magnesium sulfate. After reaction, the solvent was distilled off in a vacuum of a water-jet pump, and the precipitated powder was recrystallized from chloro­form to give white crystals of 2,2-di­chloro-3,3-dieth­oxy-1-(4-fluoro­phen­yl)propan-1-ol (yield: 2.72 g, 88%; m.p. 485–487 K). ^1^H NMR (300 MHz, DMSO-*d*_6_): δ 1.22 (2CH_3_, 6H), 3.63–3.94 (2CH_2_, 4H), 4.77 (*s*, 1H), 5.03 (*d*, *J* = 5.0 Hz, 1H), 6.42 (*d*, *J* = 5.0 Hz, 1H), 7.19 (*m*, 2H), 7.55 (*m*, 2H). ^13^C NMR (75 MHz, DMSO-*d*_6_): δ 15.51, 62.33, 79.35, 95.15, 113.45, 115.64, 127.98, 134.18, 160.28.

## 7. Refinement

Crystal data, data collection and structure refinement details are summarized in Table 2 ▸. The positions and displacement parameters of the H atoms have been refined.

## Supplementary Material

Crystal structure: contains datablock(s) I. DOI: 10.1107/S2056989025002154/oi2015sup1.cif

Supporting information file. DOI: 10.1107/S2056989025002154/oi2015Isup4.cml

Structure factors: contains datablock(s) I. DOI: 10.1107/S2056989025002154/oi2015Isup4.hkl

our response to reviewer's. DOI: 10.1107/S2056989025002154/oi2015sup3.docx

CCDC reference: 2429365

Additional supporting information:  crystallographic information; 3D view; checkCIF report

Additional supporting information:  crystallographic information; 3D view; checkCIF report

## Figures and Tables

**Table 1:** Hydrogen-bond geometry (Å, °)

*D*—H⋯*A*	*D*—H	H⋯*A*	*D*⋯*A*	*D*—H⋯*A*
O1—H1⋯O2^i^	0.82 (5)	2.17 (5)	2.975 (2)	165 (4)
O1—H1⋯O3^i^	0.82 (5)	2.43 (5)	3.046 (2)	133 (4)

Symmetry code: (i) 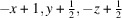.

**Table 2:** Experimental details

Crystal data	
Chemical formula	C_13_H_17_Cl_2_FO_3_
*M*_r_	311.16
Crystal system, space group	Orthorhombic, *P*2_1_2_1_2_1_
Temperature (K)	100
*a*, *b*, *c* (Å)	10.5608 (3), 10.8048 (3), 12.8872 (4)
*V* (Å^3^)	1470.52 (7)
*Z*	4
Radiation type	Cu *K*α
μ (mm^−1^)	4.10
Crystal size (mm)	0.15 × 0.12 × 0.08

Data collection	
Diffractometer	Rigaku XtaLAB Synergy Dualflex diffractometer with a HyPix detector
Absorption correction	Multi-scan (*CrysAlis PRO*; Rigaku OD, 2022 ▸)
*T*_min_, *T*_max_	0.611, 0.810
No. of measured, independent and observed [*I* > 2σ(*I*)] reflections	15942, 3123, 3111
*R*_int_	0.032
(sin θ/λ)_max_ (Å^−1^)	0.634

Refinement	
*R*[*F*^2^ > 2σ(*F*^2^)], *wR*(*F*^2^), *S*	0.025, 0.065, 1.05
No. of reflections	3123
No. of parameters	241
H-atom treatment	All H-atom parameters refined
Δρ_max_, Δρ_min_ (e Å^−3^)	0.17, −0.18
Absolute structure	Flack *x* determined using 1296 quotients [(*I*^+^) − (*I*^−^)]/[(*I*^+^) + (*I*^−^)] (Parsons *et al.*, 2013 ▸)
Absolute structure parameter	−0.001 (6)

Computer programs: *CrysAlis PRO* (Agilent, 2014 ▸), *SHELXT2014* (Sheldrick, 2015*a* ▸), *SHELXL2019* (Sheldrick, 2015*b* ▸), *DIAMOND* (Brandenburg, 1999 ▸) and *WinGX* (Farrugia, 2012 ▸).

## supplementary crystallographic information

### 2,2-Dichloro-3,3-diethoxy-1-(4-fluorophenyl)propan-1-ol Crystal data

**Table:**

C_13_H_17_Cl_2_FO_3_	*D*_x_ = 1.405 Mg m^−^^3^
*M**_r_* = 311.16	Cu *K*α radiation, λ = 1.54184 Å
Orthorhombic, *P*2_1_2_1_2_1_	Cell parameters from 13434 reflections
*a* = 10.5608 (3) Å	θ = 3.4–77.6°
*b* = 10.8048 (3) Å	µ = 4.10 mm^−^^1^
*c* = 12.8872 (4) Å	*T* = 100 K
*V* = 1470.52 (7) Å^3^	Block, colourless
*Z* = 4	0.15 × 0.12 × 0.08 mm
*F*(000) = 648	

### 2,2-Dichloro-3,3-diethoxy-1-(4-fluorophenyl)propan-1-ol Data collection

**Table:**

Rigaku XtaLAB Synergy Dualflex diffractometer with a HyPix detector	3111 reflections with *I* > 2σ(*I*)
Radiation source: micro-focus sealed X-ray tube	*R*_int_ = 0.032
ω scans	θ_max_ = 77.8°, θ_min_ = 5.3°
Absorption correction: multi-scan (CrysAlis PRO; Rigaku OD, 2022)	*h* = −12→13
*T*_min_ = 0.611, *T*_max_ = 0.810	*k* = −13→11
15942 measured reflections	*l* = −15→16
3123 independent reflections	

### 2,2-Dichloro-3,3-diethoxy-1-(4-fluorophenyl)propan-1-ol Refinement

**Table:**

Refinement on *F*^2^	Hydrogen site location: difference Fourier map
Least-squares matrix: full	All H-atom parameters refined
*R*[*F*^2^ > 2σ(*F*^2^)] = 0.025	*w* = 1/[σ^2^(*F*_o_^2^) + (0.0332*P*)^2^ + 0.5017*P*] where *P* = (*F*_o_^2^ + 2*F*_c_^2^)/3
*wR*(*F*^2^) = 0.065	(Δ/σ)_max_ < 0.001
*S* = 1.05	Δρ_max_ = 0.17 e Å^−^^3^
3123 reflections	Δρ_min_ = −0.18 e Å^−^^3^
241 parameters	Extinction correction: SHELXL2019 (Sheldrick, 2015*b*), Fc^*^=kFc[1+0.001xFc^2^λ^3^/sin(2θ)]^-1/4^
0 restraints	Extinction coefficient: 0.0022 (4)
Primary atom site location: structure-invariant direct methods	Absolute structure: Flack *x* determined using 1296 quotients [(I+)-(I-)]/[(I+)+(I-)] (Parsons *et al.*, 2013)
Secondary atom site location: difference Fourier map	Absolute structure parameter: −0.001 (6)

### 2,2-Dichloro-3,3-diethoxy-1-(4-fluorophenyl)propan-1-ol Special details

**Table:**

Geometry. All esds (except the esd in the dihedral angle between two l.s. planes) are estimated using the full covariance matrix. The cell esds are taken into account individually in the estimation of esds in distances, angles and torsion angles; correlations between esds in cell parameters are only used when they are defined by crystal symmetry. An approximate (isotropic) treatment of cell esds is used for estimating esds involving l.s. planes.

### 2,2-Dichloro-3,3-diethoxy-1-(4-fluorophenyl)propan-1-ol Fractional atomic coordinates and isotropic or equivalent isotropic displacement parameters (Å^2^)

**Table:**

	*x*	*y*	*z*	*U*_iso_*/*U*_eq_	
Cl1	0.21324 (5)	0.29037 (5)	0.37004 (4)	0.02959 (15)	
Cl2	0.30012 (5)	0.13688 (5)	0.19928 (5)	0.03094 (15)	
F1	−0.11669 (14)	0.50329 (16)	0.00613 (12)	0.0394 (4)	
O1	0.38547 (17)	0.48111 (15)	0.26820 (14)	0.0308 (4)	
H1	0.430 (4)	0.531 (4)	0.236 (3)	0.067 (13)*	
O2	0.46588 (15)	0.13111 (14)	0.38223 (12)	0.0255 (3)	
O3	0.55686 (14)	0.25502 (14)	0.26145 (12)	0.0240 (3)	
C1	0.4609 (2)	0.2489 (2)	0.33530 (17)	0.0236 (4)	
H1A	0.469 (3)	0.315 (3)	0.388 (2)	0.032 (7)*	
C2	0.3356 (2)	0.2697 (2)	0.27532 (18)	0.0239 (4)	
C3	0.3432 (2)	0.3840 (2)	0.20378 (18)	0.0249 (4)	
H3	0.410 (3)	0.363 (3)	0.149 (2)	0.033 (8)*	
C4	0.2194 (2)	0.41543 (19)	0.15014 (17)	0.0245 (4)	
C5	0.1336 (2)	0.4959 (2)	0.19664 (19)	0.0266 (4)	
H5	0.153 (3)	0.530 (3)	0.261 (2)	0.024 (7)*	
C6	0.0200 (2)	0.5259 (2)	0.14852 (19)	0.0279 (5)	
H6	−0.044 (3)	0.580 (3)	0.177 (2)	0.036 (8)*	
C7	−0.0049 (2)	0.4741 (2)	0.05275 (19)	0.0301 (5)	
C8	0.0770 (2)	0.3949 (2)	0.00310 (18)	0.0319 (5)	
H8	0.055 (3)	0.361 (3)	−0.068 (3)	0.043 (8)*	
C9	0.1903 (2)	0.3664 (2)	0.05271 (18)	0.0292 (5)	
H9	0.248 (3)	0.310 (3)	0.021 (2)	0.031 (7)*	
C10	0.4336 (2)	0.1240 (3)	0.49060 (18)	0.0328 (5)	
H10A	0.477 (3)	0.194 (3)	0.528 (2)	0.033 (7)*	
H10B	0.339 (4)	0.135 (4)	0.500 (3)	0.055 (10)*	
C11	0.4728 (3)	−0.0001 (3)	0.5306 (2)	0.0356 (6)	
H11A	0.436 (3)	−0.064 (3)	0.492 (3)	0.038 (8)*	
H11B	0.452 (3)	−0.004 (3)	0.604 (3)	0.050 (9)*	
H11C	0.565 (3)	−0.008 (3)	0.520 (3)	0.046 (9)*	
C12	0.67911 (19)	0.2820 (2)	0.30512 (18)	0.0253 (4)	
H12A	0.709 (3)	0.210 (3)	0.349 (2)	0.029 (7)*	
H12B	0.671 (3)	0.361 (3)	0.350 (3)	0.041 (8)*	
C13	0.7697 (2)	0.3031 (2)	0.2167 (2)	0.0314 (5)	
H13A	0.854 (3)	0.315 (3)	0.243 (2)	0.035 (8)*	
H13B	0.741 (3)	0.376 (3)	0.180 (3)	0.048 (9)*	
H13C	0.772 (3)	0.230 (3)	0.172 (2)	0.040 (8)*	

### 2,2-Dichloro-3,3-diethoxy-1-(4-fluorophenyl)propan-1-ol Atomic displacement parameters (Å^2^)

**Table:**

	*U*^11^	*U*^22^	*U*^33^	*U*^12^	*U*^13^	*U*^23^
Cl1	0.0233 (2)	0.0294 (3)	0.0361 (3)	0.0038 (2)	0.0084 (2)	0.0058 (2)
Cl2	0.0267 (3)	0.0218 (2)	0.0443 (3)	−0.0002 (2)	−0.0073 (2)	−0.0079 (2)
F1	0.0330 (8)	0.0470 (9)	0.0381 (8)	0.0109 (7)	−0.0091 (6)	0.0037 (7)
O1	0.0322 (9)	0.0209 (7)	0.0392 (9)	−0.0089 (7)	−0.0052 (7)	0.0006 (7)
O2	0.0293 (7)	0.0232 (7)	0.0242 (7)	−0.0001 (6)	0.0035 (6)	−0.0002 (6)
O3	0.0170 (7)	0.0280 (8)	0.0270 (7)	−0.0020 (6)	0.0015 (6)	−0.0020 (6)
C1	0.0221 (10)	0.0223 (10)	0.0265 (10)	−0.0008 (8)	0.0042 (8)	−0.0024 (8)
C2	0.0201 (9)	0.0209 (10)	0.0305 (10)	−0.0015 (8)	0.0032 (8)	−0.0032 (8)
C3	0.0219 (9)	0.0246 (10)	0.0281 (10)	−0.0031 (8)	0.0013 (8)	−0.0019 (9)
C4	0.0226 (10)	0.0231 (10)	0.0278 (10)	−0.0020 (8)	0.0026 (8)	0.0022 (8)
C5	0.0291 (11)	0.0217 (10)	0.0290 (11)	−0.0016 (9)	0.0012 (9)	−0.0002 (9)
C6	0.0274 (11)	0.0242 (10)	0.0323 (11)	0.0034 (9)	0.0029 (9)	0.0036 (9)
C7	0.0260 (11)	0.0321 (12)	0.0320 (11)	0.0014 (9)	−0.0015 (9)	0.0090 (10)
C8	0.0330 (12)	0.0359 (13)	0.0270 (12)	0.0035 (10)	−0.0006 (9)	−0.0007 (10)
C9	0.0275 (11)	0.0325 (11)	0.0275 (10)	0.0020 (10)	0.0027 (9)	−0.0008 (9)
C10	0.0310 (12)	0.0444 (14)	0.0229 (11)	0.0053 (11)	0.0023 (9)	0.0021 (10)
C11	0.0378 (14)	0.0410 (14)	0.0281 (12)	−0.0037 (11)	0.0002 (10)	0.0050 (10)
C12	0.0194 (10)	0.0248 (10)	0.0316 (10)	−0.0036 (8)	−0.0001 (8)	−0.0024 (9)
C13	0.0221 (11)	0.0347 (12)	0.0374 (12)	−0.0030 (9)	0.0039 (9)	0.0053 (10)

### 2,2-Dichloro-3,3-diethoxy-1-(4-fluorophenyl)propan-1-ol Geometric parameters (Å, º)

**Table:**

Cl1—C2	1.792 (2)	C6—C7	1.380 (4)
Cl2—C2	1.778 (2)	C6—H6	0.97 (3)
F1—C7	1.362 (3)	C7—C8	1.374 (4)
O1—C3	1.410 (3)	C8—C9	1.392 (3)
O1—H1	0.82 (5)	C8—H8	1.01 (4)
O2—C1	1.410 (3)	C9—H9	0.96 (3)
O2—C10	1.440 (3)	C10—C11	1.495 (4)
O3—C1	1.392 (3)	C10—H10A	1.01 (3)
O3—C12	1.438 (3)	C10—H10B	1.01 (4)
C1—C2	1.549 (3)	C11—H11A	0.94 (3)
C1—H1A	0.99 (3)	C11—H11B	0.97 (4)
C2—C3	1.544 (3)	C11—H11C	0.99 (4)
C3—C4	1.518 (3)	C12—C13	1.505 (3)
C3—H3	1.02 (3)	C12—H12A	1.01 (3)
C4—C5	1.391 (3)	C12—H12B	1.03 (3)
C4—C9	1.397 (3)	C13—H13A	0.97 (3)
C5—C6	1.389 (3)	C13—H13B	0.97 (4)
C5—H5	0.93 (3)	C13—H13C	0.98 (3)
			
C3—O1—H1	112 (3)	C8—C7—C6	123.3 (2)
C1—O2—C10	117.08 (18)	C7—C8—C9	117.7 (2)
C1—O3—C12	113.31 (17)	C7—C8—H8	120 (2)
O3—C1—O2	108.00 (16)	C9—C8—H8	122 (2)
O3—C1—C2	105.90 (17)	C8—C9—C4	121.2 (2)
O2—C1—C2	112.15 (17)	C8—C9—H9	119.4 (18)
O3—C1—H1A	111.9 (18)	C4—C9—H9	119.4 (18)
O2—C1—H1A	110.7 (18)	O2—C10—C11	108.5 (2)
C2—C1—H1A	108.2 (18)	O2—C10—H10A	108.3 (18)
C3—C2—C1	111.72 (17)	C11—C10—H10A	112.6 (17)
C3—C2—Cl2	109.13 (16)	O2—C10—H10B	110 (2)
C1—C2—Cl2	109.77 (14)	C11—C10—H10B	110 (2)
C3—C2—Cl1	110.15 (15)	H10A—C10—H10B	108 (3)
C1—C2—Cl1	107.12 (15)	C10—C11—H11A	111 (2)
Cl2—C2—Cl1	108.90 (11)	C10—C11—H11B	108 (2)
O1—C3—C4	112.01 (18)	H11A—C11—H11B	113 (3)
O1—C3—C2	105.07 (18)	C10—C11—H11C	108 (2)
C4—C3—C2	113.98 (17)	H11A—C11—H11C	105 (3)
O1—C3—H3	110.7 (17)	H11B—C11—H11C	111 (3)
C4—C3—H3	109.4 (17)	O3—C12—C13	107.75 (19)
C2—C3—H3	105.5 (18)	O3—C12—H12A	109.9 (16)
C5—C4—C9	118.8 (2)	C13—C12—H12A	110.4 (16)
C5—C4—C3	120.3 (2)	O3—C12—H12B	108.3 (18)
C9—C4—C3	120.9 (2)	C13—C12—H12B	110.6 (18)
C6—C5—C4	121.1 (2)	H12A—C12—H12B	110 (2)
C6—C5—H5	119.4 (17)	C12—C13—H13A	110.0 (19)
C4—C5—H5	119.6 (17)	C12—C13—H13B	107 (2)
C7—C6—C5	118.0 (2)	H13A—C13—H13B	111 (3)
C7—C6—H6	117.2 (19)	C12—C13—H13C	109.7 (18)
C5—C6—H6	124.9 (19)	H13A—C13—H13C	107 (3)
F1—C7—C8	119.0 (2)	H13B—C13—H13C	112 (3)
F1—C7—C6	117.8 (2)		
			
C12—O3—C1—O2	−80.7 (2)	O1—C3—C4—C5	−28.8 (3)
C12—O3—C1—C2	158.94 (17)	C2—C3—C4—C5	90.3 (2)
C10—O2—C1—O3	145.47 (19)	O1—C3—C4—C9	149.9 (2)
C10—O2—C1—C2	−98.2 (2)	C2—C3—C4—C9	−91.0 (2)
O3—C1—C2—C3	−49.1 (2)	C9—C4—C5—C6	1.1 (3)
O2—C1—C2—C3	−166.62 (17)	C3—C4—C5—C6	179.8 (2)
O3—C1—C2—Cl2	72.14 (18)	C4—C5—C6—C7	−0.5 (3)
O2—C1—C2—Cl2	−45.4 (2)	C5—C6—C7—F1	179.7 (2)
O3—C1—C2—Cl1	−169.77 (14)	C5—C6—C7—C8	−0.1 (4)
O2—C1—C2—Cl1	72.67 (18)	F1—C7—C8—C9	−179.8 (2)
C1—C2—C3—O1	−52.0 (2)	C6—C7—C8—C9	−0.1 (4)
Cl2—C2—C3—O1	−173.54 (14)	C7—C8—C9—C4	0.7 (4)
Cl1—C2—C3—O1	66.95 (19)	C5—C4—C9—C8	−1.2 (3)
C1—C2—C3—C4	−174.97 (18)	C3—C4—C9—C8	−179.9 (2)
Cl2—C2—C3—C4	63.5 (2)	C1—O2—C10—C11	−167.2 (2)
Cl1—C2—C3—C4	−56.0 (2)	C1—O3—C12—C13	−172.42 (18)

### 2,2-Dichloro-3,3-diethoxy-1-(4-fluorophenyl)propan-1-ol Hydrogen-bond geometry (Å, º)

**Table:**

*D*—H···*A*	*D*—H	H···*A*	*D*···*A*	*D*—H···*A*
O1—H1···O2^i^	0.82 (5)	2.17 (5)	2.975 (2)	165 (4)
O1—H1···O3^i^	0.82 (5)	2.43 (5)	3.046 (2)	133 (4)

Symmetry code: (i) −*x*+1, *y*+1/2, −*z*+1/2.
