# Supplementary material for: Synthesis, crystal structure and Hirshfeld surface analysis of 2,2-di­chloro-3,3-dieth­oxy-1-(4-fluoro­phen­yl)propan-1-ol
Source: Acta Crystallogr E Crystallogr Commun. 2025 Apr 29;81(Pt 5):444–7. doi: 10.1107/S2056989025002154 (PMC12054764; doi:10.1107/S2056989025002154)
Supplement: Supplementary file 4 [file e-81-00444-sup3.docx]

**Response to reviewers**

**Dr. Alebel N. Belay**

College of Science,

Department of Chemistry,

Bahir Dar University, Bahir Dar, P.O.Box 79, Ethiopia

E-mail: alebel.nibret@bdu.edu.et

**05 March 2025**

Manuscript ID: oi2015.

Dear Editor and Reviewers,

Thank you very much for your quick response and valuable comments. We agree with all comments and have revised the manuscript accordingly (change made highlighted in yellow; see attached Our Responses_oi2015). Below is a point-by-point response to the reviewer’s comment. If you require any further information, please do not hesitate to contact me.

Once again thank you for your time and kind consideration.

We look forward to hearing from you soon.

Sincerely,

Alebel N. Belay on behalf of all authors

**Referee 1**

The work of Guseynova and coauthors describes the synthesis and structure of the 2,2-dichloro-3,3-diethoxy-1-(4-fluorophenyl)propan-1-ol, it was done properly and I have only a few minor comments.

Add

_atom_sites_solution_primary and _atom_sites_solution_secondary. Added details

Correct values for

_computing_data_collection are indicated as 'CrysAlisPro (Agilent 2014)'	what else?

_computing_cell_refinement,

_computing_data_reduction.

The molecule shows intramolecular non-conventional H-bonds between C5-H5..O1 and C10-H10b...Cl1 with 2.820 (3) and 3.326 (3) A distance between the donor and acceptor atom, respectively.

It is my personal opinion, but I do not see a sense in discussing such hydrogen bonds unless there is an additional conformation from a complementary technique.

We do not understand this observation. These are interactions that affect the packing. Figure 4 (a), finger plot. Interaction-type labeling is quite useless on the finger plot with all contacts, either expand the figure for different types of contacts or remove it.

**Response:** The expanded fingerprint plot has been incorporated in the revised manuscript.

**Review from Referee 2**

Referee 2 attached the following notes to their review.

The manuscript from Guseynova *et al*. reports on the crystal structure and Hirshfeld surface analysis of 2,2-dichloro-3,3-diethyoxy-1-(4-fluorophenyl)propan-1-ol. Considering the versatility of the synthetic applications involving the halogenation of the ketones, this study offers valuable insights into the structural properties of the compound, as well as the simple method for the separation of the two chiral isomers. In general, the analysis of single crystal X-ray diffraction data and the refinement process are well-documented. Moreover, the thorough and detailed discussion about each computational and experimental result will be helpful for readers to follow the authors’ explanation. In light of these considerations, we recommend that this manuscript be suitable for publication after the authors address the following minor revisions:

**Minor Revisions**

(1) There is a typo on line 42; ‘crystallization’ should be corrected to “crystallization”.

**Response:** corrected

(2) It is interesting that the two chiral isomers can be separated through crystallization. Can the authors please provide more information on the crystallization conditions, such as the solvent used and the temperature at which the process was carried out?

**Response:** The text was modified

**Review from Referee 3**

Referee 3 attached the following notes to their review.

The manuscript by Guseynova and coworkers reports the synthesis, crystal structure, and Hirshfeld surface analysis of 2,2-dichloro-3,3-diethoxy-1-(4-fluorophenyl)propan-1-ol. The authors have completed a rather thorough Hirshfeld analysis of the title compound, including a description of the various interaction energies at play. This work should be suited for publication in Acta Crystallographica Section E after some issues are first addressed. Detailed comments and questions follow below.

**Major Comments**

It is not reasonable to have a *T*Max value = 1.00, as this does not make physical sense since there should always be some amount of absorption, even if it is very small. This may be due to the absorption correction, but can the authors please explain this further?

**Response:** This was an oversight. The data is now correct as 0.81

Please address the following comments and questions related to the novelty of this work:

While the introduction briefly lists the importance of α-halo ketones at the start, the authors only briefly mention the point of understanding halogen bond donor centers in hydrogenation products for improving functional properties. It is not clear to this reviewer what kind of functional properties are of particular interest here and how the halogen bond donor centers in particular affect these properties. Adding a little extra information here would be very helpful. Furthermore, what makes the particular target compound important for these sorts of studies?

**Response:** The text was modified “… we believe that the halogen atoms in hydrogenation products of ketones can act as the halogen bond donor centers in crystal engineering.”

Section 5 lists very few fragments that could be useful for comparison to the title compound. It would greatly help the discussion to highlight some of the key differences between their target compound and other hydrogenation products with halogen bond donor centers. Especially if there are any other Hirshfeld surface analyses that could provide a valuable comparison to the title compound.

**Response:** Some additional structures have been included (references Essa et al, 2013, and 2015)

The Hirshfeld surface analysis and information on the intermolecular interactions provide additional insights into the structure of the target compound. However, there are a few points that can be clarified.

While the Hirshfeld analysis study helps to quantify the percentage of each interaction type present, it is not clear if any general conclusions can be drawn from this work. For example, is this distribution typical for such compounds?

**Response:** From this analysis, it can be observed that the total polar interactions (Cl...F + Cl...H + F...H + O...H) are 40.2 %, whereas the non-polar and van der Waals interaction total is 59.1 %. Therefore, the molecule has balanced polar and non-polar interactions, which indicates an amphiphilic nature of the compound.

The authors mention in the abstract there is no *π-*stacking interaction (lines 17-18).

However, the opposite is stated in the Hirshfeld analysis section (lines 79-82).

**Response:** We are sorry for the confusion. After a careful investigation of the compound's crystal structure, we found that the molecule does not have an π-stacking interaction. However, the appearance of the H...C interactions from the fingerprint plot is due to the O-H...C(OEt)_2_ interactions, which are given below.

Figure 5 is a bit confusing, as it is not clear what the difference is between (a), (b), and (c) visually apart from the color. The three colors signify Eele, Edis, and Etot, but it is difficult to see any differences between the three in the figure itself.

**Response:** Thank you very much for the comments. Apart from the color, the vertical and diagonal lines in Figures 5a and 5b are distinctly different. The line in 5b is much thicker than the one in 5a. However, all the tubes and lines (horizontal, vertical, and diagonal) are bolder than those of 5a and 5b. For clarity, the images are given below.

**Minor Comments**

- There are some typos and grammatical errors that should be fixed.

**Response:** The text has been checked and corrected

Some of the references do not appear to be formatted correctly, as noted in the reference checking results. This may be because the journals do not follow the citation format with a final page number, but please ensure that this is the only reason why.

**The following references may be incorrectly formatted/ Response:**

Guseinov, F. I., Malinnikov, V. M., Lialin, K. N., Kobrakov, K. I., Shuvalova, E. V., Nelyubina, Y. V., Ugrak, B. I., Kustov, L. M. & Mahmudov, K. T. (2022). *Dyes Pigments*, **197**, 109898.
[*Unrecognized journal title. Missing final page numbers?*] This journal reports the number of papers with no number of pages

Guseinov, F. I., Pistsov, M. F., Movsumzade, E. M., Kustov, L. M., Tafeenko, V. A., Chernyshev, V. V., Gurbanov, A. V., Mahmudov, K. T. & Pombeiro, A. J. L. (2017). *Crystals*, 7, 327.
[*Missing final page numbers?*] This journal reports the number of paper with no number of pages

Yang, S. & Fang, X. (2023). *Tetrahedron* **145**, 133609.
[*Missing final page numbers?*] This journal reports the number of paper with no number of pages

Mahesha, Udaya Kumar, A. H., Vindya, K. G., Pampa, K. J., Rangappa, K. S., Lokanath, N. K. (2022). *J. Mol. Struct*. **1250** 131746.
[*Author surname with no initials? Missing final page numbers?*] No initial report for “Mahesha “ in the pdf of the paper; this journal reports the number of the paper with no number of pages

Biswas, G., Afzal, M., Halder, U. C. & Sepay, N. A. (2025). *J. Mol. Struct*. **1322**, 140358.
[*Missing final page numbers?*] This journal reports the number of the papers with no number of pages

Sepay, N., Mandal, A. & Chakraborty, A. (2023). *J. Organomet. Chem*. **983**, 122556.
[*Missing final page numbers?*] This journal reports the number of papers with no number of pages

Das, S., Samanta, S., Mandal, A. & Sepay, N. (2023). *J. Mol. Struct*. **1292**, 136136.
[*Missing final page numbers?*] This journal reports the number of papers with no number of pages
